# Supplementary material for: De Novo Asp219Val Mutation in Cardiac Tropomyosin Associated with Hypertrophic Cardiomyopathy
Source: Int J Mol Sci. 2022 Dec 20;24(1):18. doi: 10.3390/ijms24010018 (PMC9820293; doi:10.3390/ijms24010018)
Supplement: Supplementary file 1 [file ijms-24-00018-s001.zip › ijms-2076631-supplementary.pdf]

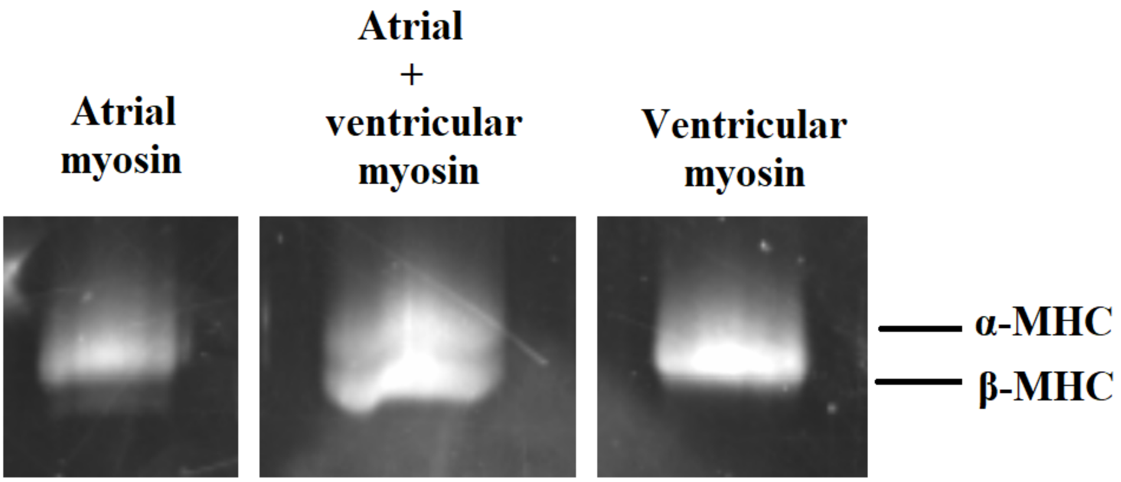

Figure S1. Example of gel electrophoresis of myosin heavy chain isoforms. The gel was stained by SYPRO Ruby (Thermo Fisher Scientific, USA). The gel was imaged on the ChemiDoc MP Imaging System (Bio-Rad, USA), and band densities were determined with Image Lab 5.2.1 software (Bio-Rad, USA). Atrial myosin contained ~95±1% α-MHC, 5±1% β-MHC, and ventricular myosin contained 100% β-MHC.

An example of gel electrophoresis of ventricular (*1*) and atrial (*2*) myosin from a pig's heart is shown in Figure 2. Unlike the atrial and ventricular light chains of other animals (rabbit, rat, etc.), the atrial and ventricular light chains of the pig's heart in gel run close to each other (Svensson C, Morano I, Arner A. In vitro motility assay of atrial and ventricular myosin from pig. J Cell Biochem. 1997 Nov 1;67(2):241-7).

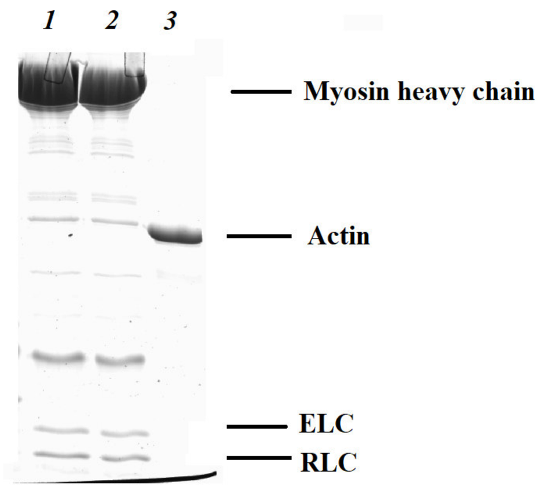

Figure S2. Example of gel electrophoresis of ventricular (*1*) and atrial (*2*) myosin from pig's heart and rabbit skeletal actin (*3*). Gel was stained by Coomassie Blue R and G (1:1). The gel was imaged on the Densitometer GS-800 (Bio-Rad, USA).
